# Contribution of MicroRNAs in Chemoresistance to Cisplatin in the Top Five Deadliest Cancer: An Updated Review

**DOI:** 10.3389/fphar.2022.831099

**Published:** 2022-04-04

**Authors:** Pía Loren, Nicolás Saavedra, Kathleen Saavedra, Nadine De Godoy Torso, Marília Berlofa Visacri, Patricia Moriel, Luis A. Salazar

**Affiliations:** ^1^ Center of Molecular Biology and Pharmacogenetics, Scientific and Technological Bioresource Nucleus, Universidad de La Frontera, Temuco, Chile; ^2^ School of Medical Sciences, University of Campinas, Campinas, Brazil; ^3^ Faculty of Pharmaceutical Sciences, University of Campinas, Campinas, Brazil

**Keywords:** microRNA, drug-resistance, cisplatin, sensitivity, cancer

## Abstract

Cisplatin (DDP) is a well-known anticancer drug used for the treatment of numerous human cancers in solid organs, including bladder, breast, cervical, head and neck squamous cell, ovarian, among others. Its most important mode of action is the DNA-platinum adducts formation, inducing DNA damage response, silencing or activating several genes to induce apoptosis; these mechanisms result in genetics and epigenetics modifications. The ability of DDP to induce tumor cell death is often challenged by the presence of anti-apoptotic regulators, leading to chemoresistance, wherein many patients who have or will develop DDP-resistance. Cancer cells resist the apoptotic effect of chemotherapy, being a problem that severely restricts the successful results of treatment for many human cancers. In the last 30 years, researchers have discovered there are several types of RNAs, and among the most important are non-coding RNAs (ncRNAs), a class of RNAs that are not involved in protein production, but they are implicated in gene expression regulation, and representing the 98% of the human genome non-translated. Some ncRNAs of great interest are long ncRNAs, circular RNAs, and microRNAs (miRs). Accumulating studies reveal that aberrant miRs expression can affect the development of chemotherapy drug resistance, by modulating the expression of relevant target proteins. Thus, identifying molecular mechanisms underlying chemoresistance development is fundamental for setting strategies to improve the prognosis of patients with different types of cancer. Therefore, this review aimed to identify and summarize miRs that modulate chemoresistance in DDP-resistant in the top five deadliest cancer, both *in vitro* and *in vivo* human models.

## 1 Introduction

Globally, cancer is the first leading cause of death. In 2020, 19.3 million new cases of cancer and almost 10 million deaths from cancer (Ferlay et al., 2021; Sung et al., 2021). Cisplatin [cis-diamminedichloroplatinum (II), DDP], discovered by Rosenberg and his colleagues in 1965 (Rosenberg et al., 1969), was the first platinum compound approved by FDA for cancer treatment in the United States in 1978 (FDA, 1978). It is a well-known chemotherapeutic drug used for the treatment of numerous human cancer in solid organs, including head and neck, testis, small cells and non-small cells lung cancer, ovarian, cervical, and bladder. Once DDP crosses the cytosol, the low concentration of chloride present triggers two hydrolyses of the DDP, forming positively charged DDP derivative, which binds to negatively charged DNA bases, inducing DNA damage by forming DNA-platinum adducts, and simultaneously initiating self-defense mechanisms to activate or silence multiple genes, resulting in DNA damage response and repair pathways (Hu et al., 2016), cell cycle arrest (Velma et al., 2016) and DDP-induced apoptosis (Tanida et al., 2012). However, treatment response to DDP differs, and the main problem to its effectiveness is the development of drug resistance (Amable, 2016; Ko and Li, 2019). Cisplatin-resistance is inferred mainly when the usual clinical dose of DDP is magnified in drug-intensive therapy protocols and may require cytotoxic concentrations as much as 50–100-fold in addition to those needed for sensitive cells (Siddik, 1999). In fact, any factor that influences those processes can lead to the development of resistance to DDP. Moreover, drug resistance is responsible for over 90% of deaths in cancer patients receiving traditional chemotherapeutic drugs (Bukowski et al., 2020). Besides, the epithelial-mesenchymal transition (EMT) process contributes to chemoresistance by transforming epithelial cells into mesenchymal cells and altering cell-cell adhesion as well as the cellular extracellular matrix, leading to invasion of tumor cells (Ashrafizadeh et al., 2020). Autophagy, a process which degrades and recycles cellular proteins and organelles in response to cellular stresses, has been shown to attenuate the sensitivity of therapeutic drugs, protecting cancerous cells from death (Li W. et al., 2019). Thus, there is a crucial necessity to comprehend the underlying molecular mechanisms and recognize strategies to counteract DDP and facilitate predictions of the clinical response to therapy.

Non-coding RNAs are molecules that regulate gene expression under physiological and pathological conditions (Virginie et al., 2019) and are further divided into two principal groups, small ncRNAs (shorter than 200 bp) and long ncRNA (longer than 200 bp). MicroRNAs a class of small ncRNAs, are a kind of short-chain, linear, approximately 21–25 nucleotides long that negatively regulate gene targets the post-transcriptional level by perfect complementarity of their “seed” region to 3′-UTR of its target mRNA, inducing their degradation. If there is a mismatch or imperfect complementarity, it results in translational repression (Guo et al., 2019). The latest release of the miRbase database (v22) contains 2654 human mature miRs sequences (Kozomara et al., 2019), which confirms their importance on gene expression regulation. Not surprisingly, atypical expression and/or activity of ncRNAs can affect the outcome of cancer treatment and allow tumors to acquire drug-resistant phenotypes (Yo et al., 2012; Tang et al., 2020). An increasing number of studies have shown that ncRNAs play an essential role in several types of cancer and miRs have been associated with DDP resistance, making them important potential therapeutic targets. So, in this narrative review, we summarize the current literature on the contribution of miRs that modulate chemoresistance to DDP in the top five deadliest cancer reported in 2020, some strategies to sensitize DDP-cells and reduce their malignant capacities, both *in vitro* and *in vivo* human models.

## 2 The Top Five Deadliest Cancer

**FIGURE 1:**
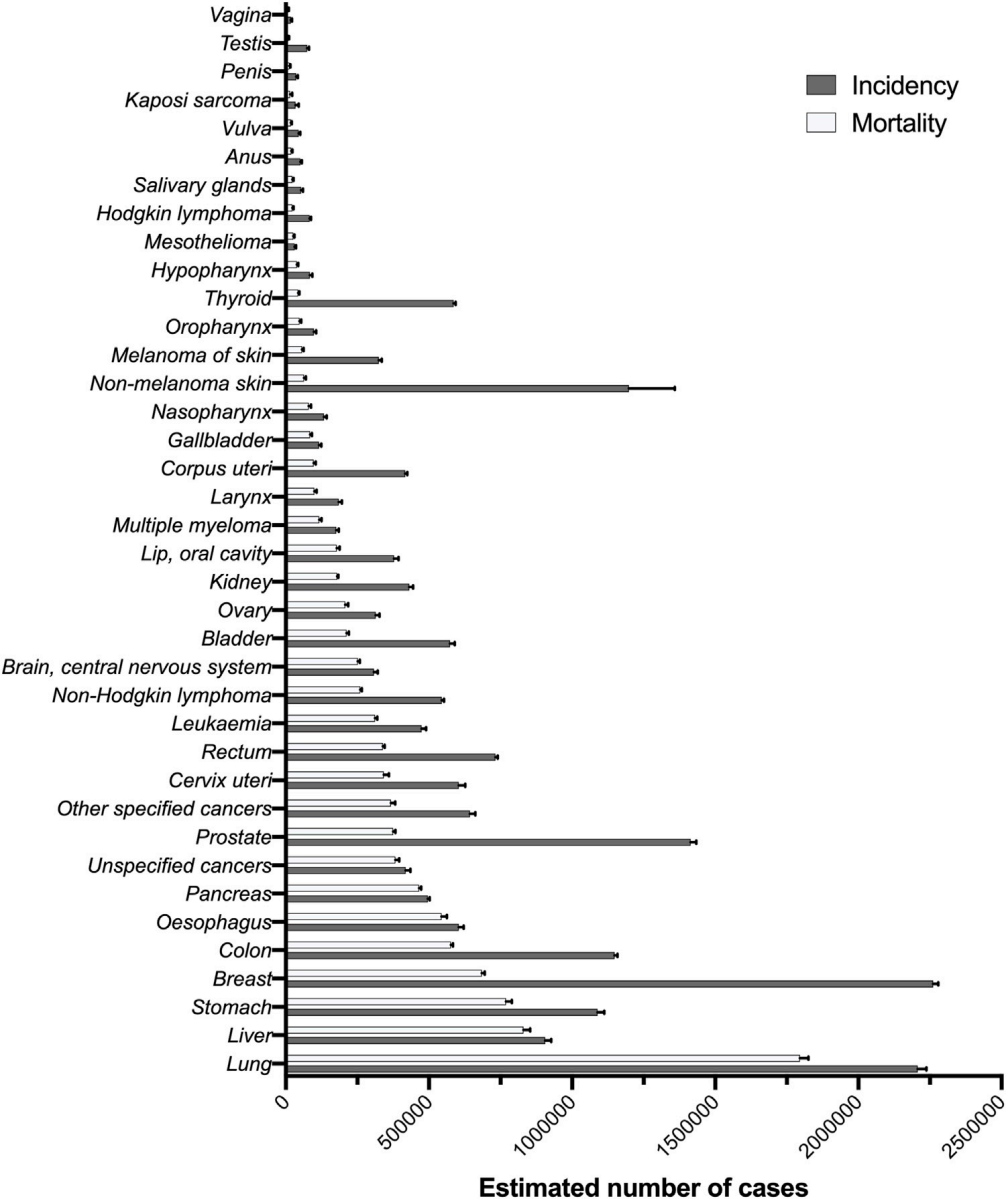
Estimated number of new cancer cases and cancer deaths worldwide for 2020 (Ferlay et al., 2021; Sung et al., 2021).

The most common cause of cancer death for about 13% of total cancer diagnoses remains by far lung cancer (Ferlay et al., 2021; Sung et al., 2021). The global incidence of lung cancer estimated in 2020 was approximately 2206800 new cases and 1796100 cancer deaths (Figure 1). In terms of clinical and tumor genetics, lung cancer can be divided into small and non-small cell lung cancer. Non-small cell lung cancer (NSCLC) represents about 80%–95% of all diagnosed lung cancer cases, and NSCLC remains the leading cause of cancer death worldwide. The efficacy of DDP-based chemotherapy in cancer is limited by the occurrence of innate and acquired drug resistance and acquired resistance of NSCLC cells against cisplatin is the consequence of altered signaling leading to reduced G2/M cell cycle arrest and apoptosis (Sarin et al., 2017). On the other hand, small-cell lung cancer (SCLC) is a distinct form of lung cancer with unique clinical and histological characteristics, representing 10%–15% of all new cases of lung cancer, and SCLC cancer tends to grow and spread faster than NSCLC (Kitamura et al., 2009). SCLC is highly sensitive to the initial cycle of chemotherapy and, in many cases chemotherapy-resistant SCLC emerges, leading to rapid patient mortality. DDP-resistance in lung cancer can be induced by alterations to a huge number of intracellular pathways, where miRNAs play a vital role (Table 1), even though very few studies have demonstrated the role of miR on DDP-resistance in SCLC.

**TABLE 1:** MicroRNAs involved in DDP-chemoresistance in lung cancer.

miR	Target	Model	Expression	References
miR-1	ATG3	*in vitro*: A-549 & NCI-H1299 cells	Down	Hua et al. (2018)
		*in vivo*: 30 DDP-resistant non-small cell lung cancer patient samples		
miR-7	Bcl-2	*in vitro*: SPC-A1 cells	Down	Cheng et al. (2017)
		*in vivo*: 108 paired of resected tumors from lung adenocarcinoma patients		
miR-10a	PIK3CA	*in vitro*: A-549 & NCI-H1299 cells	Up	Huang T. et al. (2020)
		*in vivo*: Blood samples from 6 lung adenocarcinoma patients		
	STAT3, STAT5	*in vitro*: A-549/DDP cells	Up	Sun et al. (2015)
miR-15b	PEBP4	*in vitro*: A-549/DDP cells	Up	Zhao et al. (2015)
		*in vivo*: 30 tissues collected from patients with advanced lung adenocarcinoma		
miR-17	ATG7	*in vitro*: A-549/DDP & NCI-H1299/DDP cells	Down	Huang FX et al. (2019)
miR-18a	IRF-2	*in vitro*: NCI-H226/DDP & A-549/DDP cells	Up	Xiao and He, (2020)
		*in vivo*: Lung biopsies of 85 non-small cell lung cancer patients		
	PTEN	*in vitro*: A-549/DDP	Up	Xiao et al. (2018)
		*in vivo*: 40 non-small cell lung cancer tissue samples		
miR-19a	PTEN	*in vitro*: A-549/DDP & NCI-H460/DDP cells	Up	Xiao et al. (2020)
		*in vitro*: 68 subjects diagnosed with non-small cell lung cancer		
miR-21	PTEN	*in vitro*: A-549/DDP cells	Up	Liang et al. (2021)
miR-25	Cyclin E2	*in vitro*: NCI-H146, NCI-H209, NCI-H446, NCI-H510A & NCI-H889 cells	Up	Zhao et al. (2014)
		*in vivo*: 9 pairs of small cell lung cancer tumor tissues		
miR-25-3p	PTEN	*in vitro*: A-549/DDP & NCI-H1299/DDP cells	Up	Sun et al. (2021a)
miR-26a	HMGA2	*in vitro*: A-549/DDP cells	Down	Yang et al. (2016)
miR-29a	REV3L	*in vitro*: A-549/DDP cells	Down	Chen et al. (2019b)
		*in vivo*: 30 non-small cell lung cancer tissues obtained from 30 patients		
miR-29b-3p	COL1A1	*in vitro*: A-549/DDP cells	Down	Jia and Wang, (2020)
		*in vivo*: 20 cases of tumor tissues		
miR-31	ABCB9	*in vitro*: SPC-A1, LTEP-a2, NCI-H460 & NCI-H1299 cells	Up	Dong et al. (2014)
miR-32	ROBO1	*in vitro*: A-549/DDP & NCI-H1299/DDP cells	Down	Zheng et al. (2021b)
		*in vivo*: Non-small cell lung cancer patient samples		
miR-34a-5p	TRIM29	*in vitro*: HCC827, NCI-H522 & NCI-H23 cells	Down	Luo et al. (2020)
		*in vivo*: 50 lung cancer specimens		
miR-96	LMO7	*in vitro*: A-549, PC-9 & NCI-H1299 cells	Up	Wu et al. (2017)
		*in vivo*: 56 lung adenocarcinoma patient specimens		
miR-98-5p	CTR1	*in vitro*: A-549/DDP cells	Up	Jiang et al. (2016)
miR-100-5p	mTOR	*in vitro*: A-549/DDP cells	Down	Qin et al. (2017)
miR-101-3p	MCL-1	*in vitro*: A-549 and NCI-H1299 cells	Down	Wang et al. (2018)
	Not reported	*in vitro*: NCI-H460, A-549 & SK-MES-1 cells	Down	Hua et al. (2021)
		*in vivo*: Samples of 25 lung cancer patients		
miR-103a-3p	NF-1	*in vitro*: A-549/DDP cells	Up	Zhu et al. (2020a)
		*in vivo*: 20 patients with non-small cell lung cancer		
miR-106a	ABCA1	*in vitro*: A-549/DDP cells	Up	Ma et al. (2015)
miR-106b-5p	PKD2	*in vitro*: A-549/DDP cells	Down	Yu et al. (2017)
miR-127-3p	MDM2	*in vitro*: A-549/DDP & NCI-H1299/DDP cells	Down	Zeng et al. (2020)
		*in vivo*: 40 non-small cell lung cancer patients who receiver surgical resection		
miR-128-2	E2F5	*in vitro*: ﻿A-549 cells	Up	Donzelli et al. (2012)
miR-130b	PTEN	*in vitro*: A-549/DDP & NCI-H446/DDP cells	Up	Zhang et al. (2018)
miR-133b	GSTP1	*in vitro*: A-549/DDP & NCI-H1299/DDP cells	Down	Lin et al. (2018)
miR-134	FOXM1	*in vitro*: A-549/DDP cells	Down	Li et al. (2017)
miR-138-5p	ATG7	*in vitro*: A-549/DDP cells	Down	Pan et al. (2019)
		*in vivo*: Samples of 60 non-small cell lung cancer subjects		
miR-140-3p	Wnt/β-catenin	*in vitro*: A-549, NCI-H1299, NCI-H292 & Calu-3 cells	Down	Wu et al. (2020b)
		*in vivo*: RNA array dataset GSE74190		
miR-142-5p	PD-L1	*in vitro*: A-549/DDP & HCC827/DDP cells	Down	Zhu et al. (2021)
		*in vivo*: Samples of 46 lung adenocarcinoma patients		
miR-144-3p	Not reported	*in vitro*: A-549C/DDP & NCI-H460/DDP cells	Down	Tian et al. (2019)
		*in vivo*: 54 tissue samples from patients with non-small cell lung cancer		
miR-145	CDK6	*in vitro*: Calu-6 & Calu-6/DDP cells	Up	Bar et al. (2015)
		*in vivo*: Primary tumors of patients with non-small cell lung cancer		
	KLF4	*in vitro*: A-549/DDP cells	Down	Cui et al. (2018)
		*in vivo*: 52 tumor tissue samples from patients with non-small cell lung cancer		
	Not reported	*in vitro*: A-549/DDP cells	Down	Zhang et al. (2019)
miR-146a	JNK-2	*in vitro*: A-549/DDP cells	Down	Pang et al. (2017)
	NF-κB1	*in vitro*: A-549/DDP & Calu-1/DDP	Down	Jiang et al. (2017a)
		*in vivo*: 28 non-small cell lung cancer tissues from patients		
miR-148b	DNMT1	*in vitro*: A-549/DDP & SPC-A1/DDP cells	Down	Sui et al. (2015)
miR-149-5p	DCLK1	*in vitro*: A-549/DDP & NCI-H1299/DDP cells	Down	Zhan et al. (2020)
		*in vivo*: 70 samples of patients with non-small cell lung cancer		
miR-152	Bcl-2, NF-κB	*in vitro*: A-549 & A-549/DDP cells	Down	Zhao et al. (2019)
miR-182-5p	GLI2	*in vitro*: NCI-H460/DDP & A-549/DDP cells	Down	Seidl et al. (2020)
		*in vivo*: 27 lung adenocarcinoma tissue		
miR-185-5p	ABCC1	*in vitro*: A-549/DDP cells	Down	Pei et al. (2016)
miR-186-5p	SIX1	*in vitro*: A-549/DDP & NCI-H1299/DDP cells	Down	Liu et al. (2020b)
		*in vivo*: Samples of 50 non-small cell lung cancer patients		
miR-195-5p	CHEK1	*in vitro*: A-549 & NCI-H1299 cells	Down	Zuo et al. (2019)
miR-196a	Not reported	*in vitro*: A-549/DDP cells	Up	Li et al. (2016a)
miR-200a	β-catenin	*in vitro*: A-549/DDP cells	Down	Tang et al. (2020)
miR-200b	Bcl-2, XIAP	*in vitro*: A-549/DDP cells	Down	Sun et al. (2012)
miR-200c	Bcl-2, XIAP	*in vitro*: A-549/DDP cells	Down	Zhu et al. (2012a)
	No Reported	*in vitro*: Calu-1, NCI-H520, SK-MES-1, H596, Calu-3, NCI-H522, NCI-H1395, NCI-H1299 & NCI-H460 cells lines	Down	Ceppi et al. (2010)
		*in vivo*: Non-small cell lung cancer patient samples		
miR-200c-3p	ERCC3, ERCC4	*in vitro*: SGC-7901/DDP cells	Down	Li et al. (2019a)
miR-202	KRAS	*in vitro*: NCI-H441 & A-549 cells	Down	Sun et al. (2018)
		*in vivo*: 25 primary non-small cell lung cancer tumor tissues		
miR-202-5p	P-gp	*in vitro*: A-549/DDP cells	Down	Shen et al. (2020a)
miR-206	MET	*in vitro*: A-549/DDP & NCI-H1299/DDP cells	Down	Chen et al. (2016b)
		*in vivo*: 34 lung adenocarcinoma tissues		
miR-216b	Beclin-1	*in vitro*: A-549/DDP cells	Down	Chen et al. (2019a)
miR-217	LHPP	*in vitro*: A-549/DDP cells	Up	Yang et al. (2021)
miR-219a-5p	FGF9	*in vitro*: A-549/DDP & SPC-A1/DDP cells	Down	Rao et al. (2019)
		*in vivo*: Tumor tissues collected from 81 non-small cell lung cancer patients		
miR-223	FBXW7	*in vitro*: A-549, NCI-H358 & NCI-H1299 cells	Up	Wang et al. (2020b)
miR-326	WNT2B	*in vitro*: NCI-H358, A-549, NCI-H1299 & NCI-H1650 cells	Down	Wu et al. (2020c)
		*in vivo*: 180 tumors from patients with non-small cell lung cancer		
miR-330-5p	DCLK1	*in vitro*: A-549 & NCI-H1299/DDP cells	Down	Ge et al. (2021)
		*in vivo*: DDP-resistant non-small cell lung cancer tissues		
miR-377-3p	GOT1	*in vitro*: A-549/DDP, NCI-H1299/DDP & Calu-3/DDP cells	Down	Zhu et al. (2020b)
miR-381	ID-1	*in vitro*: A-549, A-549/DDP & NCI-H460 cells	Down	Huang et al. (2018)
miR-383	RBM24	*in vitro*: A-549/DDP cells	Down	He et al. (2021)
		*in vivo*: 93 Lung adenocarcinoma tissues		
miR-429	Bcl-2, XIAP	*in vitro*: A-549/DDP cells	Down	Zhu et al. (2012a)
miR-432	E2F3, AXL	*in vitro*: A-549 & NCI-H1299 cells	Down	Chen et al. (2016a)
		*in vivo*: human lung adenocarcinoma samples		
miR-448	SATB1	*in vitro*: A-549/DDP cells	Down	Ning et al. (2020)
		*in vivo*: 146 patients diagnosed with lung cancer		
miR-451	Not reported	*in vitro*: A-549 cells	Down	Bian et al. (2011)
		*in vivo*: Non-small cell lung cancer patient samples		
	TRIM66	*in vitro*: A-549/DDP & H157/DDP cells	Down	Wang et al. (2019a)
		*in vivo*: 30 non-small cell lung cancer tumor tissues		
miR-454-3p	STAT3	*in vitro*: A-549/DDP & H157/DDP cells	Down	Zhao et al. (2018)
miR-486-5p	TWF1	*in vitro*: A-549/DDP cells	Down	Jin et al. (2019)
		*in vivo*: 46 patient samples with non-small cell lung cancer		
miR-493	TCRP1	*in vitro*: A-549/DDP cells	Down	Gu et al. (2017)
		*in vivo*: Non-small cell lung cancer patient samples		
miR-497	Bcl-2	*in vitro*: A-549/DDP cells	Down	Zhu et al. (2012b)
miR-503	FANCA	*in vitro*: A-549, NCI-H446, NCI-H1650 & NCI-H1299 cells	Down	Li et al. (2014)
		*in vivo*: 65 samples of non-small cell lung cancer patients		
miR-514a-3p	ULK1	*in vitro*: A-549/DDP cells	Down	Shen et al. (2020b)
		*in vivo*: 30 DDP-resistant & 30 DDP-sensitive non-small cell lung cancer tissues		
miR-519	ZBTB5	*in vitro*: SK-MES-1, NCI-H1299/DDP and A-549/DDP cells	Down	Tang et al. (2019)
		*in vivo*: 40 lung cancer tissue samples		
miR-548a	ROBO1	*in vitro*: A-549/DDP & NCI-H1299/DDP cells	Down	Zheng et al. (2021b)
		*in vivo*: Non-small cell lung cancer patient samples		
miR-556-5p	NLRP3	*in vitro*: A-549/DDP & NCI-H1299/DDP cells	Up	Shi et al. (2021)
		*in vivo*: 47 non-small cell lung cancer patients		
miR-630	Bcl-2	*in vitro*: NCI-H358, NCI-H23, A-549, NCI-H1299, TL-4 & CL1-0 cells	Down	Chen et al. (2018)
		*in vivo*: 114 patients with non-small cell lung cancer		
miR-641	HOXA9	*in vitro*: A-549/DDP, NCI-H1299/DDP & Calu-6/DDP cells	Down	Zhao et al. (2020b)
miR-1236-3p	ATG7	*in vitro*: NCI-H522/DDP & A-549/DDP cells	Down	Wang et al. (2020c)
		*in vivo*: 31 lung cancer tissues		
miR-1244	TP53	*in vitro*: A-549/DDP cells	Down	Li et al. (2016b)
miR-1269b	PTEN	*in vitro*: A-549/DDP cells	Up	Yang et al. (2020a)
		*in vivo*: 32 lung tissue samples obtained from non-small cell lung cancer patients		
miR-4443	METTL3	*in vitro*: A-549/DDP &NCI-H460 cells	Up	Song et al. (2021)
		*in vivo*: Non-small cell lung cancer tumor tissue		
miR-4458	REV3L	*in vitro*: A-549/DDP & NCI-H460/DDP cells	Down	Pang et al. (2020)
		*in vivo*: 30 pairs of sensitive tissues and resistant samples		
miR-4701-5p	NFE2L1	*in vitro*: PC-9, Calu-3, A-549 & HCC827 cells	Down	Wei et al. (2020)
		*in vivo*: 40 lung adenocarcinoma tissues		
let-7i	BAG-1	*in vitro*: A-549/DDP cells42	Down	Sun et al. (2017)
		*in vivo*: Lung adenocarcinoma tissue samples		

Followed by lung cancer, the second cause of cancer death is due to liver cancer (Ferlay et al., 2021; Sung et al., 2021). Liver cancer comprises a heterogeneous group of malignant liver tumors with different histological features and an unfavorable prognosis (Anwanwan et al., 2020). The global incidence of liver cancer estimated in 2020 was approximately 905700 new cases and 830200 cancer deaths (Figure 1). The prognosis for liver cancer is poor, due to merely 5%–15% of patients are eligible for surgical removal, because of diminished hepatic regenerative capacity (Anwanwan et al., 2020). Treatment options for more advanced stages include chemotherapy, however, fewer than one-third of patients benefit from the treatment, and drug resistance is evident within 6 months of initiating the regimen (El-Serag et al., 2008). On this basis, miRs have been involved in DDP-resistance in lung cancer and will be listed in Table 2.

**TABLE 2:** MicroRNAs involved in DDP-chemoresistance of liver cancer.

miR	Target	Model	Expression	References
miR-9-5p	EIF5A2	*in vitro*: Hep 3B2.1-7, Hep-G2, SNU-387 & SNU-449 cells	Down	Bao et al. (2020)
miR-29c	SIRT1	*in vitro*: Hep-G2/DDP cells	Down	Zhang and Luo, (2018)
miR-30a	Beclin-1	*in vitro*: Hep-G2 cells	Down	Zou et al. (2012)
miR-31-5p	MAGEA3	*in vitro*: Hep-G2 & Huh-7 cells	Down	Chen et al. (2020)
miR-33a-5p	Not reported	*in vitro*: Hep 3B2.1-7/DDP & MHCC97-L/DDP	Down	Meng et al. (2017)
miR-103	NOR1	*in vitro*: Hep3B2.1-7/DDP& Huh-7/DDP cells	Up	Luo et al. (2019)
		*in vivo*: 120 hepatocellular carcinoma tissues		
miR-155-5p	PDK1	*in vitro*: Hep3 B2.1-7/DDP & Huh-7/DDP	Down	Li et al. (2021c)
		*in vivo*: Samples of 82 hepatocellular carcinoma tumor tissue		
miR-340-5p	NRF2	*in vitro*: SMMC-7721/DDP, HuH7/DDP and Hep-G2/DDP cells	Down	Wu et al. (2019)
		*in vivo*: 30 hepatocellular carcinoma tissues		
miR-326	RUNX2	*in vitro*: Hep-G2 & Huh-7 cells	Down	Guan et al. (2020)
		*in vivo*: Hepatocellular carcinoma patient samples		

Followed by liver cancer, the third cause of cancer death is due to stomach cancer (Ferlay et al., 2021; Sung et al., 2021). Gastric cancer is one of the most commonly diagnosed malignancies. The global incidence of stomach cancer estimated in 2020 was approximately 1089100 new cases and 768800 cancer deaths (Figure 1). In recent years, a rising body of evidence has revealed that miRs are dysregulated in almost all types of tumors, including gastric, modulating the proliferation, stemness, tumor immune escape, invasion, angiogenesis, and drug resistance of tumor cells (Chen et al., 2021). Some studies in which miRs play a major role in mediating DDP-resistance in stomach cancer will be detailed in Table 3.

**TABLE 3:** MicroRNAs involved in DDP-chemoresistance of stomach cancer.

miR	Target	Model	Expression	References
miR-21	PI3K, Akt, mTOR	*in vitro*: AGS/DDP cells	Up	Gu et al. (2020)
	PI3K/Akt	*in vitro*: MGC-803 cells	Up	Zheng et al. (2017)
	Not reported	*in vivo*: 67 samples of gastric cancer patients	Up	Qi et al. (2017)
	PTEN	*in vitro*: SGC-7901/DDP cells	Up	Yang et al. (2013)
miR-25	FOXO3a	*in vitro*: SGC-7901/DDP cells	Up	He et al. (2017)
miR-30a	LC3-I, LC3-II	*in vitro*: SGC-7901 & SGC-7901/DDP cells	Down	Du et al. (2018)
miR-34a	ABCB1, ABCC1, ABCG2	*in vitro*: SGC-7901/DDP & MGC-803/DDP cells	Down	Cheng et al. (2018)
miR-34c	E2F1	*in vitro*: SGC-7901 cells	Down	Zheng et al. (2020)
		*in vivo*: 74 cases of paired gastric cancer tissues		
miR-95-3p	EMP1	*in vitro*: SGC-7901/DDP & AGS/DDP cells	Up	Ni et al. (2021)
		*in vivo*: Biopsies of 32 advanced gastric cancer patients		
miR-99a-5p	MTMR3	*in vitro*: BGC-823/DDP & SGC-7901/DDP cells	Down	Sun et al. (2020a)
		*in vivo*: 60 gastric cancer tissues		
miR-106a	PTEN, PI3K/Akt	*in vitro*: SGC-7901 & SGC-7901/DDP cells	Up	Fang et al. (2013)
miR-122	ERCC1	*in vitro*: MKN74 cells	Down	Song et al. (2019)
		*in vivo*: 60 samples collected from 60 gastric cancer patient		
miR-126	VEGFA, PIK3R2	*in vitro*: SGC-7901/DDP & BGC-823/DDP cells	Down	Yan et al. (2016)
		*in vivo*: 30 primary gastric cancer		
miR-129	P-gp	*in vitro*: BGC-823/DDP & MKN45/DDP cells	Down	Lu et al. (2017)
		*in vivo*: 25 gastric cancer tissues		
miR-138	FOXC1	*in vitro*: NCI-N87/DDP & AGS/DDP cells	Down	Sun et al. (2021b)
miR-138-5p	ERCC1, ERCC4	*in vitro*: SGC-7901/DDP cells	Down	Ning et al. (2019)
miR-142-3p	ROCK2	*in vitro*: AGS, SGC-7901, MKN45 & BGC-823 cells	Up	Peng et al. (2020)
		*in vivo*: 100 gastric cancer tissues from patients		
miR-144-3p	UBE2D1	*in vitro*: AGS/DDP & MKN45/DDP cells	Down	Li et al. (2021b)
		*in vivo*: Samples of 51 gastric cancer patients		
miR-182-5p	Not reported	*in vitro*: SGC-7901/DDP & BGC-823/DDP cells	Down	Huang XX. et al. (2020)
		*in vivo*: Tissues of 105 gastric cancer patients		
miR-187	TGF-β/p-SMAD4	*in vitro*: SGC-7901/DDP cells	Down	Zhu et al. (2019)
miR-192-5p	ERCC3, ERCC4	*in vitro*: SGC-7901/DDP cells	Down	Xie et al. (2019)
miR-198	PIK3R1	*in vitro*: SGC-7901/DDP & BGC-823/DDP	Down	Huang Z. et al. (2019)
		*in vivo*: 149 gastric cancer tissues		
miR-200c	ZEB2	*in vitro*: SGC-7901/DDP cells	Down	Jiang et al. (2017b)
		*in vivo*: 50 gastric cancer tissues		
miR-216a-5p	Bcl-2	*in vitro*: SGC-7901/DDP cells	Down	Zhao et al. (2020a)
		*in vivo*: Tissues from 106 patients with stage II/III gastric cancer		
miR-299-3p	EndoPDI	*in vitro*: AGS/DDP & HGC-27/DDP cells	Down	Yang et al. (2020b)
		*in vivo*: DDP-resistant and DDP-sensitive gastric cancer tissues from 35 patients		
miR-325-3p	GITR	*in vitro*: MKN45 & AGS cells	Down	Sun et al. (2020b)
		*in vivo*: 137 tissues from gastric cancer patients		
miR-362	CYLD, NF-κB	*in vitro*: SGC-7901, BGC-823, HGC-27, MKN28 & MGC-803 cells	Up	Xia et al. (2014)
		*in vivo*: 10 freshly collected gastric cancer tissues		
miR-362-5p	SUZ12	*in vitro*: SGC-7901/DDP cells	Down	Wei et al. (2019)
miR-363	FBW7	*in vitro*: MGC-803 & HGC-27 cells	Up	Zhang et al. (2016)
		*in vivo*: 71 gastric cancer samples		
miR-372	FOXO3a	*in vitro*: MGC-803/DDP & MKN28/DDP cells	Up	Wang et al. (2020a)
miR-421	E-cadherin, caspase-3	*in vitro*: AGS, MKN28, MKN45, NCI-N87, HGC-27, SNU-16 & SGC-7901 cells	Up	Ge et al. (2016)
		*in vivo*: 107 specimens of primary gastric adenocarcioma		
miR-490-3p	HMGA2	*in vitro*: BGC-823/DDP & SGC-7901/DDP cells	Down	Xia et al. (2021)
		*in vivo*: Primary gastric cancer samples obtained from 100 patients		
miR-497-5p	ATG14	*in vitro*: BGC-823/DDP & SGC-7901/DDP	Down	Song et al. (2020)
		*in vivo*: 30 gastric cancer patients		
miR-503	E2F2	*in vitro*: SGC-7901, MKN45, BGC-823, HGC-27, MFC & SGC-7910/DDP	Down	Jiang et al. (2020)
miR-505	CYLD	*in vitro*: BG-C823/DDP & SGC-7901/DDP cells	Up	Wang et al. (2020d)
miR-513a-3p	CYP1B1	*in vitro*: AGS & NCI-N87 cells	Down	Cheng et al. (2021)
		*in vivo*: 53 gastric cancer tumor tissues		
miR-574-3p	ZEB1	*in vitro*: SGC-7901/DDP cells	Down	Wang et al. (2019b)
miR-618	Bcl-2	*in vitro*: BGC-823/DDP & SGC-7901/DDP cells	Down	Zhang et al. (2020a)
		*in vivo*: 92 gastric cancer tissues		
miR-876-3p	TMED3	*in vitro*: SGC-7901/DDP & MKN45/DDP cells	Down	Peng et al. (2019)
		*in vivo*: Gastric cancer tissue samples collected from 50 patients		
miR-3619-5p	TBL1XR1	*in vitro*: AGS/DDP & NUGC-3/DDP cells	Down	Wu et al. (2020a)
		*in vivo*: Gastric cancer tissues from DDP-resistant and DDP-sensitive patients		

After stomach cancer, the fourth cause of cancer mortality is due to breast cancer (Ferlay et al., 2021; Sung et al., 2021). Breast cancer is the most commonly diagnosed cancer worldwide and female breast cancer is the most commonly diagnosed cancer (Sung et al., 2021). The global incidence estimated in 2020 was approximately 2261400 new cases and 685000 cancer deaths due to breast cancer (Figure 1). Cisplatin is currently the most effective drug used to treat breast cancer; however, DDP-resistance presents a major challenge in the successful treatment of breast cancer. Breast cancer can be invasive or non-invasive. Invasive breast cancer is cancer that spreads into adjacent tissues and/or distant organs, while non-invasive breast cancer does not go beyond the milk ducts or lobules in the breast (Beikman et al., 2013). Some studies in which miRs play a major role in mediating DDP-resistance in breast cancer will be detailed on Table 4.

**TABLE 4:** MicroRNAs involved in DDP-chemoresistance of breast cancer.

miR	Target	Model	Expression	References
miR-133a	FTL	*in vitro*: MCF-7/DDP cells	Down	Chekhun et al. (2013)
miR-141-3p	KLF12	*in vitro*: MCF-7 & MDA-MB-231 cells	Up	Zhou et al. (2021)
		*in vivo*: 62 patients with breast cancer diagnosed		
miR-199b-5p	PXN	*in vitro*: MDA-MB-231, Hs 578T, HCC 1806, HCC1599 & CAL-51 cells	Down	Du et al. (2020)
miR-203	SOCS3	*in vitro*: MCF-7, ZR-75 & MDA-MB-231 cells	Up	Ru et al. (2011)
		*in vivo*: 10 specimens from breast cancer patients		
miR-218	BRCA1	*in vitro*: MCF-7 & MCF-7/DDP cells	Down	He et al. (2015)
		*in vivo*: BRC patient samples		
miR-381	MDR1	*in vitro*: MCF-7/DDP & MDA-MB-231/DDP cells	Down	Yi et al. (2019)
		*in vivo*: 46 tumor tissue specimens		
	Not reported	*in vitro*: MCF-7/DDP & MDA-MB-231/DDP cells	Down	Mi et al. (2020)
		*in vivo*: 42 tumor tissues obtained from breast cancer patients		
miR-1307	MDM4	*in vitro*: MCF-7/DDP and MDA-MB-468/DDP cells	Down	Wang and Zhu, (2018)

The last cause of cancer mortality is due to colorectal cancer. Colorectal cancer starts when normal cells in the lining of the colon or rectum change and grow out of control, forming a mass called a tumor (Weitz et al., 2005). The global incidence of colon and rectum cancer estimated in 2020 was approximately 1880700 new cases and 915900 cancer deaths (Figure 1). The relative survival rate for colorectal cancer is 64% at 5-year following diagnosis and 58% at 10 years (Siegel et al., 2020). This can be determined by resistance to DDP, which may compromise the efficacy of chemotherapy, and some miRs related are described in Table 5.

**TABLE 5:** MicroRNAs involved in DDP-chemoresistance of colorectal cancer.

miR	Target	Model	Expression	References
miR-125b-5p	HK2	*in vitro*: HT29, SW620, HCT 116, SW480 & DLD-1 cells	Down	Shi et al. (2020)
		*in vivo*: 35 colorectal cancer patients		
miR-137	Not reported	*in vitro*: SW480, HT-29, SW620 and LoVo cells	Down	Zheng et al. (2021a)
miR-148a	WNT10b	*in vitro*: SW480/DDP cells	Down	Shi et al. (2019)
		*in vivo*: 90 colorectal cancer specimens		
miR-155	FOXO3	*in vitro*: SW620 cells	Up	Gao et al. (2018)
		*in vivo*: Samples from patients with colorectal cancer		
miR-487a-3p	SOX9	*in vitro*: HT29, SW480, SW620 & HCT 116 cells	Up	Sun et al. (2020c)
		*in vivo*: 6 colorectal cancer tumor tissues		
miR-490-3p	TNKS2	*in vitro*: SW480, LoVo, DLD-1, SW48, RKO, HCT 116, HT29, SW620 & HCT 8 cells	Down	Li et al. (2021a)
		*in vivo*: Samples of 162 colorectal cancer patients		
miR-497	Bcl-2	*in vitro*: HCT 8/DDP cells	Down	Zheng et al. (2021c)
	IGF1-R	*in vitro*: HCT 116, LoVo, COLO 205, SW480 & SW620 cells	Down	Guo et al. (2013)
		*in vivo*: Colorectal cancer patient samples		
miR-526b-3p	KLF12	*in vitro*: HCT 116/DDP and LoVo/DDP/DDP cells	Down	Zhang et al. (2021)
		*in vivo*: Colorectal cancer tissue obtained from 37 patients		
miR-593-5p	CCND1	*in vitro*: SW1463, HR-8348 & SW837 cells	Down	Qu et al. (2020)
miR-645	SOX30	*in vitro*: Caco-2, LIM1215, COLO 205, SW620, HCT 116, SW480, LIM 1863, EB & WiDr cells	Up	Guo et al. (2017)
		*in vivo*: Colorectal cancer tissue		
miR-4486	ATG7	*in vitro*: HCT 116/DDP & SW480/DDP cells	Down	Wang et al. (2021c)

## 3 MicroRNAs Involved in DDP-Chemoresistance

### 3.1 MicroRNAs Involved in Cell Cycle

The cell cycle is a highly and complex mechanism that ensures complete and accurate cell division, and it is driven by several proteins named CDKs, which in turn are positively regulated by cyclins (A-E) and negatively regulated by CDKIs (1-9) (Sherr and Roberts, 1999); and also by MDMs (Flores and Sobrevia, 2000). Cells can acquire drug resistance due to a relative insensitivity to a chemotherapeutic agent because of the position of the cells in the cell cycle (Shah and Schwartz, 2001). Mechanistically, some miRs can target multiple proteins involved in cell cycle progression. Cisplatin-resistant colorectal cancer cell lines, HCT 116/DDP and LoVo/DDP, have been shown to express low levels of miR-526-3p, compared with parental cells, and silencing of miR-526b-3p increases cyclin D1 expression and reduced cell cycle arrest, promoting thus DDP-resistance in colorectal cancer cells enhances (Zhang et al., 2021). Mechanistically, miR-526-3p targeted KLF12. This is important, due to KLF12 is able to regulate cell death, by promoting the cell cycle transition through S phase and therefore cell proliferation and reduced expression levels of KLF12 results in increased ability of lung cancer cells to form tumors *in vivo* (Godin-Heymann et al., 2016). Another miRs involved in cell cycle is miR-25. High levels of miR-25 are found in several lung cancer cells (Zhao et al., 2014). Also, tumor-derived cells express elevated cyclin E2 levels, which accelerated cell cycle in G1 stage (Gudas et al., 1999). However, downregulation of miR-25 is able to induce cell cycle arrest by reducing CDK2 and cyclin E2 expression in lung cancer cells, thus sensitizing cells to DDP (Zhao et al., 2014). Roundabout guidance receptor 1 (ROBO1), a cancer-promoting oncogene, has been negatively correlated with the prognosis of patients, due to ROBO1 promotes the genesis and progression of cancer metastasis (Li et al., 2013). On this basis, miR-32 and miR-548a have proved to target the 3′-UTR sequence of ROBO1, promoting ROBO1 expression and activating the Wnt/β-catenin pathway. In addition, lung cancer cells exhibit low miR-32 and miR-548a levels, leading to an enhanced ROBO1 expression and displaying a DDP-resistant phenotype in A-549 cells (Zheng J. et al., 2021). In the same way, tankyrase 1 and 2 (TNKS1/2) are regulators of Wnt signaling by controlling the activity of the β-catenin destruction complex by reducing G1 cell cycle arrest and senescence (Foronda et al., 2019). Mechanistically, TNKS2 is targeted by miR-490-3p, and its increased expression promoted the chemoresistance of colorectal cancer cells (Li J. et al., 2021).

Likewise, levels of miR-103 are upregulated hepatocellular carcinoma cells (Luo et al., 2019), while miR-200a is reduced in DDP-resistant lung cancer cells (Tang et al., 2020). Also, NOR1 was targeted by miR-103 (Luo et al., 2019). It has been demonstrated that NOR1, a tumor suppressor gene, is downregulated in NPC cells and NOR1 that enhances cancer stem-like cell properties in tumor cells by enhancing the Akt and Wnt/β-catenin pathways (Wang et al., 2017). Additionally, miR-200a targeted β-catenin, regulating negatively its expression and its downstream molecules cyclin D1 and vimentin (Tang et al., 2020). Furthermore, cyclin D1 is also directly targeted by miR-593-5p in colorectal cancer cells (Qu et al., 2020) and by miR-1296 in breast cancer cells (Albakr et al., 2021). Cyclin D1 levels must be high during G1 phase for a cell to begin DNA synthesis, but then must be reduced to low levels during S phase to allow for efficient DNA synthesis, however, an aberrant cyclin D1 activity is observed in tumor cells (Montalto and De Amicis, 2020). Additionally, enhanced cyclin D1 and surviving expression enhance resistance by reducing G1 phase arrest and apoptosis, downregulating REV3L expression and leading to enhanced cell proliferation and invasive capacity (Zhu et al., 2016). Moreover, REV3L was targeted by miR-29a and miR-4458 and high expression was observed in tumoral tissues due to a decreased expression in lung cancer cells (Chen X. et al., 2019; Pang et al., 2020). However, overexpression of miR-29a could reduce viability and proliferation and enhance DDP-induced apoptosis of A-549/DDP cells treated with 5 μg/ml DDP (Chen X. et al., 2019).

Expression of miR-203 is enhanced in breast cancer cells, and, mechanistically, miR-203 targeted SOCS3, enhancing DDP-resistance (Ru et al., 2011). However, silencing of miR-203 sensitized breast cancer cells, and it was observed that those cells displayed a higher level of p21, associating these changes with decreased chemoresistance (Ru et al., 2011). This is important, due to p21 being a type of cell cycle regulator that plays a dual role in tumor cells, regulating the cell cycle, inducing apoptosis, and inhibiting cell proliferation (Wang L. et al., 2021). MDMs are nuclear factors that regulate the cell cycle at the G1/S phase transition, whose function and expression are altered in various types of human neoplasms (Momand et al., 1998). Degradation of p21 could be mediated by MDM4, in cooperation with MDM2, leading to abrogation of G1 cell cycle arrest (Jin et al., 2008). It has been reported that miR-1307 and miR-127-3p are downregulated in DDP-resistant breast cancer and lung cancer cell, respectively, and, mechanistically, they directly targeted MDM4 and MDM2, promoting DDP-resistance (Wang and Zhu, 2018; Zeng et al., 2020).

### 3.2 MicroRNAs involved in Autophagy

Autophagy is an intracellular self-digesting process for the regulation of cell homeostasis, that occurs under several stressful conditions, including organelle damage, the presence of abnormal proteins, and nutrient deprivation (Yun and Lee, 2018). In addition, autophagy regulates the properties of cancer stem-cells by contributing to the maintenance of stemness and the development of resistance to anticancer reagents (Yun and Lee, 2018). MiRs are involved in DDP response of tumor cells by regulation of autophagy.

A key initial event in autophagy is the formation of the autophagosome, and this step is mediated by the serine/threonine protein kinase ULK1 (Zachari and Ganley, 2017). Mechanistically, ULK1 is targeted by miR-514a-3p in NSCLC cells (Shen Q. et al., 2020). Moreover, miR-514a-3p was markedly downregulated in lung tissues and cells, and autophagy was found to be promoted (Shen Q. et al., 2020).

Autophagy-related (ATG) genes are indispensable for autophagosome formation, and enhanced autophagy and proliferation, and reduced apoptosis have been related to enhanced ATGs expression in cancer cells (Wang Q. et al., 2021). In this context, miR-17, miR-138-5p and miR-1236-3p enhances autophagy activity in lung cancer cells *via* ATG7 targeting (Huang FX. et al., 2019; Pan et al., 2019; Wang et al., 2020c). In addition, miR-4486 also enhances autophagy by targeting ATG7 in colorectal carcinoma cells (Wang W. et al., 2021). ATG3 is another key gene involved in autophagy and it is targeted by miR-1 in NSCLC cells (Hua et al., 2018). It has been observed that there was significant miR-1, miR-17, miR-138-5p and miR-1236-3p downregulation in NSCLC cells (Hua et al., 2018; Huang FX. et al., 2019; Pan et al., 2019; Wang et al., 2020c). Likewise, miR-4486 was also decreased in colorectal cancer cells (Wang W. et al., 2021). Moreover, miR-4443 is also upregulated in lung cancer cells. Besides, METTL3 was confirmed as a direct target gene of miR-4443 (Song et al., 2021). METTL3, a m6A methyltransferase, is able to regulate autophagy by increasing the critical genes, such as ATG5 and ATG7 (Liu S. et al., 2020). In this way, enhanced ATG7 levels promote the conversion of LC3-I into LC3-II and improve Beclin-1 expression, supporting autophagy and chemoresistance of lung cancer (Huang FX. et al., 2019; Wang et al., 2020c) and colorectal cancer cells (Wang W. et al., 2021). Beclin-1 also plays an important role in autophagy-induced tumorigenesis and drug resistance, altering cell growth, cellular microenvironment and cell division (Usman et al., 2021). Beclin-1 has been reported to be targeted by miR-30a in liver cancer cells (Zou et al., 2012) and by miR-216b in lung cancer cells (Chen L. et al., 2019), and both miRs are downregulated in both cancer types, suggesting their role in autophagy activity.

Moreover, miR-99a-5p was found to be upregulated in DDP-resistant gastric cancer cells (Sun G. et al., 2020). Mechanistically, miR-99a-5p targeted MTMR3 and enhanced MTMR was confirmed to induce autophagic activity (Taguchi-Atarashi et al., 2010), promoting resistance to chemotherapy in tumors.

### 3.3 MicroRNAs Involved in Epithelial-to-Mesenchymal Transition

The initiation of metastasis involves an increase in cell motility mediated by loss of cell-cell adhesion, caused by E-cadherin repression and augmented N-cadherin expression, in a process commonly known as epithelial-to-mesenchymal transition (EMT) (Taylor et al., 2010). In this way, high invasive potential, decreased E-cadherin expression and increased DDP-resistance has been founded in lung NCI-H1299, H596 and NCI-H522 cancer cells, due to a reduced miR-200c expression (Ceppi et al., 2010), and in liver Hep-G2 and Huh-7 cancer cells, also due to a decreased miR-31-5p expression (Chen et al., 2020).

A molecule implicated in the EMT process is polycomb ring finger (BMI1). Enhanced BMI1 expression, a known proto-oncogene, promoted EMT, augmented stemness and rendered cell drug resistance (Paranjape et al., 2014). On this basis, it has been reported that miR-802 expression is downregulated in DDP-resistant gastric cancer tissues and cells. Mechanistically, miR-802 directly targeted BMI1 and their boosted levels in gastric cancer cells promote EMT process (Liu et al., 2020d).

Zinc finger E-box binding homeobox 1 and 2 (ZEB1 and ZEB2) are transcription factors that promote tumor invasion and metastasis by inducing EMT in carcinoma cells (Zhang P. et al., 2015; DaSilva-Arnold et al., 2019). Also, ZEB1 has been founded to be targeted by miR-574-3p (Wang M. et al., 2019), while ZEB2 is targeted by miR-200c in gastric cancer cells (Jiang T. et al., 2017) and their upregulation contributed to DDP-resistance. Both miRs were founded to be downregulated in SGC-7901/DDP cells (Jiang T. et al., 2017; Wang M. et al., 2019). Even more, miR-223, miR-363 and miR-500a-3p directly targeted F-box and WD repeat domain containing 7 (FBXW7) and promote DDP-resistance in gastric cancer cells (Zhang et al., 2016; Wang et al., 2020b; Lin et al., 2020). FBXW7 (also known as FBW7) directly binds and degrades the EMT-inducing transcription factor ZEB2 in a phosphorylation-dependent manner and its loss can induce an EMT phenotype (Li N. et al., 2019). However, since miR-363 and miR-500a-3p are upregulated in gastric cancer MGC-803 and HGC-27 cells, and miR-223 in lung cancer A-549, NCI-H358 and NCI-H1299 cells, those cell lines display an EMT phenotype (Zhang et al., 2016; Wang et al., 2020b; Lin et al., 2020).

Another molecule that participated in the EMT process is doublecortin-like kinase 1 (DCLK1), a cancer stem cell marker. DCLK1 is functionally involved in maintaining cancer stemness and the process of EMT (Chandrakesan et al., 2016). Also, DCLK1 has been found to be targeted by miR-330-5p and its upregulation contributes to DDP-resistance. Likewise, miR-330-5p was found to be downregulated in lung cancer A-549 and NCI-H1299 resistant cells, promoting DDP-resistance in lung cancer cells (Ge et al., 2021).

Melanoma-associated antigen A3 (MAGEA3) enhances migration, invasion and proliferation by activation of EMT and Wnt signaling pathway in HeLa cells (Gao et al., 2020). In the same way, enhanced expression of MAGEA3 was found in drug-resistant cells (Bertram et al., 1998) and knockdown of MAGEA3 expression caused a reduction in proliferation and colony formation ability (Xie et al., 2016). Mechanistically, MAGEA3 is targeted by miR-31-5p, and its upregulation is associated with DDP-resistance. Likewise, miR-31-5p was founded to be downregulated in liver Hep-G2 and Huh-7 cancer cells, thus promoting DDP-resistance (Chen et al., 2020).

Collagen 1A1 (COL1A1) has been highly expressed and associated with poor prognosis in multiple cancers and positively correlated with the abundance of CAFs, macrophages, and tumor-infiltrating lymphocytes, and activation of EMT process (Liu et al., 2021). Also, miR-29b-3p directly target COL1A1 to promote DDP-resistance. Parallel that, it has been reported miR-29a-3p expression was reduced in lung A-549 cancer resistant cells, and augmented COL1A1 levels are associated with DDP-resistance (Jia and Wang, 2020).

Six homeobox 1 (SIX1) and Notch receptor 2 (NOTCH2) protein expressions have been associated with invasive lung cancer, by inducing EMT and thus promoting advanced malignant phenotypes (Mimae et al., 2012). Expression of miR-186-5p was downregulated lung A-549/DDP and NCI-H1299 resistant cancer cells. In addition, miR-186-5p negatively regulated SIX1 and SIX1 was upregulated in DDP resistant cancer cells (Liu X. et al., 2020).

SRY-related high mobility group-box 9 (SOX9) is a transcription factor, which acts as a proto-oncogene, implicated with the Wnt/β-catenin pathway activation and in the expression of EMT-associated proteins (Huang JQ. et al., 2019; Panda et al., 2021). Mechanistically, SOX9 is targeted by miR-487a-3p in colorectal cancer cells. Additionally, colorectal cancer cells displayed low miR-487a-3p levels, promoting SOX9 expression in colorectal HT29 and SW480 cells, exhibiting the DDP-resistant phenotype (Sun Y. et al., 2020).

Paxillin (PXN) is a cytoplasmatic protein which regulates focal adhesion. Also, PXN has been shown to promote the activation of ERK and enhance the EMT process (Wen et al., 2020). Bioinformatic analysis has proved that PXN is a direct target of miR-199b-5p. Also, decreased miR-199b-5p levels are observed in breast cancer cells, promoting the EMT process by reducing E-cadherin levels (Du et al., 2020). Loss of E-cadherin has been shown to promote the growth, invasion, and enhance drug resistance of CrC cells and, contribute to the progression and metastatic dissemination (Chen et al., 2012).

EIF5A2 plays an important role in many biological processes, including tumor formation, cancer cell growth, maintenance of cancer stem cells and EMT process (Meng et al., 2019). Bao et al. (2020) demonstrated EIF5A2 was targeted by miR-9 and was upregulated in lung tumor cells, thus promoting chemoresistance to DDP by increasing EMT process. Also, Wnt10b has been involved in enhanced tumor cell stemness by upregulation of OCT4 and NANOG expression. In colorectal cancer, WNT10b is directly targeted by miR-148a and the reduced miR-148a expression enhances Wnt10b levels to allow drug resistance in cancer therapy (Shi et al., 2019).

### 3.4 MicroRNAs Involved in Apoptosis

Apoptosis is a form of programmed cell death. In this pathway, molecular mechanisms which trigger inhibition of apoptosis responsible for DDP-resistance includes MAPK dysregulation, enhanced Bcl-2 or Bcl-XL expression, suppression of caspase-3 activity, enhanced PI3K/Akt activity, and so on (Siddik, 2003). A number of miRs have been described to be involved in the regulation of apoptosis.

Mitogen-activating protein kinases (MAPK) are molecules involved in apoptosis. There are three major MAPK pathways that involve the extracellular signal-regulated kinases: ERK1/2, JNK and p38 kinase. Chen et al., proved that MAPK3 was directly targeted by miR-206 in gastric cancer cells (Chen Z. et al., 2019). Also, Sun T. et al. (2020) demonstrated that miR-325-3p interacted with GITR, and upregulated expression contributes to DDP-resistance in gastric cancer cells. On this basis, GITR is able to enhance ERK phosphorylation, suggesting that GITR is associated whit MAPK-pathway activation (Ronchetti et al., 2004). Also, Rao et al. (2019) showed that miR-219a-5p directly targeted FGF9, and its enhanced expression leads to DDP-resistance in lung cancer cells. In this way, the low miR-325-3p and miR-219a-5p expression observed in gastric and lung cancer cells activate MAPK pathway, contributing to DDP-resistance (Ronchetti et al., 2004; Rao et al., 2019). Also, Zhou and Chen demonstrated that miR-135b interacted with MST1, and upregulated expression activates MAPK pathway, contributing to DDP-resistant phenotype (Zhou and Chen, 2019).

The intrinsic-mediated apoptotic pathway causes mitochondrial membrane potential loss, cytochrome *c* release and cleaved caspase-3. Bcl-2 is located in the mitochondrion membrane, and is related to the mitochondrial membrane potential loss and the cytochrome *c* release (Chen et al., 2015). MiR-7, miR-145, miR-146a, miR-152, miR-181b, miR-200b, miR-200c, miR-429, miR-451, miR-497 and miR-630 are reported to target Bcl-2 in lung cancer tissues and/or cells, and negatively regulate its expression (Zhu et al., 2010; Bian et al., 2011; Zhu et al., 2012a; Zhu et al., 2012b; Cheng et al., 2017; Pang et al., 2017; Chen et al., 2018; Zhang et al., 2019; Zhao et al., 2019). Likewise, miR-497 also interacts with Bcl-2 in colorectal cancer cells (Zheng ZH. et al., 2021). In this way, the low miR-7, miR-152, miR-181b, miR-200b, miR-200c, miR-429, miR-497 and miR-630 expression shown leads to decreased apoptosis incidence, resulting in a DDP-resistant phenotype in lung and colorectal cancer cells. Also, increased Bcl-2 levels have been associated with decreased cleaved-caspase 3 and E-cadherin levels, triggering EMT process and promoting DDP-phenotype (Du et al., 2020). The E2F family consists of 8 genes and 10 protein products encoded by these genes, which are crucial for regulating apoptosis, and they have been classified as transcriptional activators (E2F1-3), predicted to be oncogenic, or transcriptional repressors (E2F4-8), predicted to have tumor suppressor functions (Xie et al., 2021). E2Fs have been associated with the upregulation of Bcl-2, which contributes to uncontrolled tumor growth (Donzelli et al., 2012; Zheng et al., 2020; Zhou, 2020; Wu et al., 2021). In this context, miR-432 and miR-503 suppress E2F3 (Chen L. et al., 2016; Zhou, 2020), miR-34c targets E2F1 (Zheng et al., 2020), and miR-128-2 interacts with E2F5 (Donzelli et al., 2012), by targeting their 3′UTR mRNA. The reduced miR-432 and miR-34c expression observed in lung and gastric cancer cells were associated with advanced tumor stage and mortality and allowed E2F1 and E2F3 to be overexpressed in DDP-resistant phenotype (Chen L. et al., 2016; Zheng et al., 2020). Another molecule implicated in apoptosis is ID1. ID1 regulates p53 and NF-κB pathways, regulating Bax and Bcl-2 genes, thus providing a survival advantage under drug treatment (Kim et al., 2008). In this sense, miR-381 directly targeted ID1 and the reduced miR-381 levels observed in lung cancer cells allows an enhanced ID1 expression, reducing apoptosis and triggering a DDP-resistant phenotype (Huang et al., 2018). Finally, JNK2 negatively regulates the activity of genes related to tumor suppression and the induction of cell apoptosis (Chen et al., 2002). Regarding that, JNK2 was identified as a direct target of miR-146a and the low miR-146a levels reduced the apoptosis rate and enhanced the relative invasion rate of lung cancer cells (Pang et al., 2017).

The PI3K-Akt pathway is a major survival pathway activated in cancer. In this sense, phosphatase and tensin homolog (PTEN) is a molecule capable of inactivate the Akt signaling pathway and acts as a negative regulator of PI3K/Akt signaling (Georgescu, 2010). Also, PTEN/PI3K/Akt pathway regulates the signaling of multiple biological processes, such as apoptosis, and also enhances PI3K/Akt/mTOR pathway, conferring drug resistance and further cancer progression in breast cancer cells (Dong et al., 2021). MiR-18a, miR-19a, miR-21, miR-25-3p, miR-130b and miR-1269b in lung cancer cells (Xiao et al., 2018; Zhang et al., 2018; Xing et al., 2019; Yang W. et al., 2020; Gu et al., 2020; Sun B. et al., 2021; Liang et al., 2021), and miR-21 and miR-106a in gastric cancer cells (Fang et al., 2013; Yang et al., 2013; Zheng et al., 2017) directly regulate PTEN, and reduced miRs levels expression in cancer cells promote PTEN expression, triggering apoptosis and DDP-resistance. Additionally, HMGA2 and KLF4 regulation are able to promote PI3K/Akt phosphorylation, resulting in increased drug resistance (Deng et al., 2021), and miR-26a interacts with HMGA2 (Yang et al., 2016) and miR-145 with KLF4 (Cui et al., 2018) to promote DDP-resistance in lung cancer cells. Moreover, MET, a proto-oncogene, also activates PI3K/Akt pathway via promoting PTEN and CDKN1A expression and reducing apoptosis (Ohta et al., 2015). In this way, miR-206 regulates MET protein in A-549 lung cancer cells by directly targeting MET 3′-UTR and activated MET/PI3K/Akt/mTOR signaling pathway to induce DDP resistance (Chen QY. et al., 2016). To contribute to Akt activation and DDP-resistance, PI3K also is targeted by miR-10a (Huang T. et al., 2020). In the same way, two downstream effectors of the PI3K/Akt pathway are also regulated by miRs. Mammalian target of rapamycin (mTOR) acts as a target gene of miR-100-5p in lung cancer (Qin et al., 2017) controlling cell growth, proliferation and survival (Populo et al., 2012). Besides, FOXO3 also is regulated by miR-155 in colorectal cancer cells (Gao et al., 2018) and by miR-372 in gastric cancer cells (Wang C. et al., 2020), and enhanced FOXO3 expression by Akt promotes cell survival and resistance (Populo et al., 2012). Even more, miR-155-5p also targets PDK1 in liver cancer (Li et al., 2021c). It has been shown that PDK1 and PDK2 cause phosphorylation and activation of Akt after its translocation to inner membrane, modulating the function of numerous substrates involved in the regulation of cell survival, cell cycle progression and cellular growth (Fresno Vara et al., 2004). So, enhanced expression of miR-155-5p increases cell proliferation and reduces apoptosis of Hep3B2.1-7 liver cancer cells (Li et al., 2021c).

Other signal transductions involved in DDP-resistance are the nuclear factor (NF)-κB and apoptosis-related signaling pathways. NF-κB is known to play an important role in cell survival and inflammation. Several miRs have been reported to regulate NF-κB, such as miR-146a, miR-152 and miR-381 in lung cancer cells (Jiang P. et al., 2017; Huang et al., 2018; Zhao et al., 2019). Reduced expression of miR-146a (Jiang P. et al., 2017), miR-152 (Zhao et al., 2019) and miR-381 (Huang et al., 2018) is observed in DDP-resistant A-549 cells, which gives rise to a heightened NF-κB expression and promotes DDP-resistant phenotype. Moreover, another study demonstrated that GSTP1 was able to interact with IKKβ to activate NF-κB and induced the expression and release of IL-6, thus mediating drug resistance in breast cancer cells (Dong et al., 2020). Furthermore, miR-133b was diminished in DDP-resistant lung cells (Lin et al., 2018). Finally, miR-362 and miR-505 overexpression were observed in gastric cancer cells (Xia et al., 2014; Wang Z. et al., 2020), and their enhanced expression promoted nuclear accumulation of NF-κB/p65, due to both miRs targeted CYLD directly and its downregulation mediated NF-κB activation. Besides, Zhang and Luo found that miR-29c was downregulated in HepG2/DDP cells, and demonstrated that miR-29c targeted SIRT1 (Zhang and Luo, 2018). SIRT1 may have enhanced activity in tumor cell growth by promoting NF-κB expression (Yeung et al., 2004).

The Wnt/β-catenin signaling pathway participates in various physiological processes such as proliferation, differentiation, apoptosis, migration and invasion; on the other hand, dysregulation of the Wnt/β-catenin contributes to the development and progression of some solid tumors (Ge and Wang, 2010). Mir-130b, miR-140-3p, miR-326, and miR-1249 directly enhance the noncanonical Wnt pathway in liver and lung cancer cells (Zhang et al., 2018; Wu S. et al., 2020; Carotenuto et al., 2020; Wu Y. et al., 2020). Also, SOX30, a tumor suppressor, acts as a transcription factor by binding directly to the p53 promoter and reduces SOX30 expression, resulting in enhanced β-catenin expression and Wnt/β-catenin pathway activation (Liu et al., 2020c). Guo et al. (2017) demonstrated miR-645 directly targeted SOX30 in colorectal cancer cells, enhancing DDP-resistant phenotype.

Other molecules also have been reported to confer DDP-resistance by inhibiting apoptosis. CYP1B1, a cytochrome P450 enzyme, is overexpressed in malignant ovarian cancer (Zhu et al., 2015). MiR-513a-3p had the same binding site to CYP1B1, low miR-513a-3p levels enhance CYP1B1 expression, conferring DDP-resistance by reducing DDP-induced apoptosis in gastric cancer cells (Cheng et al., 2021). ROCK1 and ROCK2 proteins are narrowly associated with tumor progress and lymph node metastasis (Zhang J. et al., 2015). Moreover, ROCK2 was regulated by miR-142-3p, and its reduced levels enhance ROCK2 expression, resulting in a DDP-resistant phenotype by reducing DDP-induced apoptosis in gastric cancer cells (Peng et al., 2020).

### 3.5 MicroRNAs Involved in Drug Efflux

The reduced uptake of water-soluble drugs and augmented drug efflux from cancer cells are the biochemical and cytological mechanisms of drug resistance in cancer cells (Chen et al., 2015). P-glycoprotein (P-gp, also known as MDR1) is encoded by the multidrug resistance gene (ABCB1). P-gp acts as a drug pump and it can bind to several drugs and pump them out of the cells, thereby decreasing their intracellular concentration and the sensitivity of cancer cells to the drug (Breier et al., 2005). P-gp is influenced by miR-30 and miR-129 in gastric cancer cells (Lu et al., 2017; Du et al., 2018), and by miR-144-3p, miR-145, and miR-202-5p in lung cancer cells (Tian et al., 2019; Zhang et al., 2019; Shen JG. et al., 2020).

Also, two additional ABC transporters, the multidrug resistance-associated protein 1 (MRP1; encoded by ABCC1), and ABCG2 are also implicated in multidrug resistance (Robey et al., 2018). Mechanistically, ABCC1 was targeted by miR-185-5p and negatively regulated its expression in lung cancer cells (Pei et al., 2016). Additionally, miR-144-3p and miR-145 also influenced the expression of MRP1 in lung cancer cells (Tian et al., 2019; Zhang et al., 2019), and by miR-381 in breast cancer cells (Yi et al., 2019) thus contributing to DDP-resistant phenotype.

## 4 Conclusion and Future Perspectives

**FIGURE 2:**
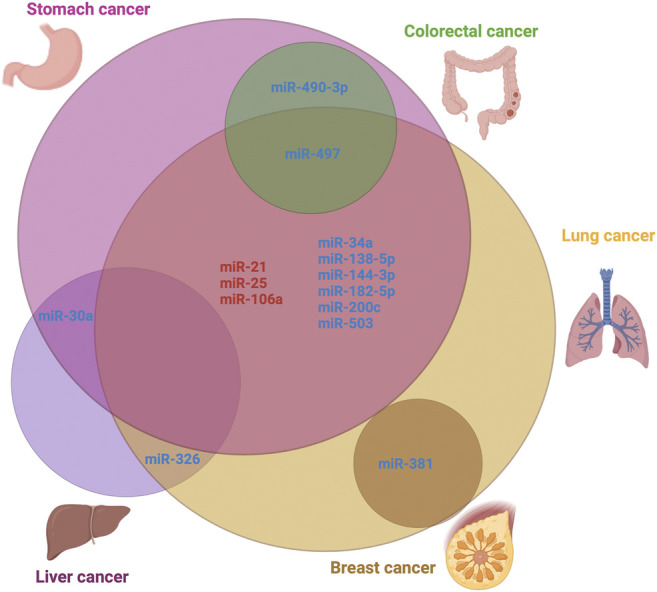
Venn diagram showing the common microRNAs expressed in the top five deadliest cancer. Upregulated microRNAs are highlighted in red color, whereas downregulated ones are in blue color.

Resistance to DDP is a major challenge that hampers the success of cancer treatment. According to current knowledge, multiple factors such as DNA damage and repair, transport process, autophagy, and apoptosis are involved in resistance to platinum-based drugs. Some dysregulated miRs functioned as an oncogenic molecules and others acted as a tumor repressor, and we tried to provide a general vision about this effect. Understanding the underlying molecular mechanisms of DDP-resistance is fundamental to reverse chemoresistance. In this way, it is possible to develop strategies to identify biomarkers of drug response and resistance, being useful in future clinical trials and rational management of cancer patients (Figure 2). Vast evidence shows that specific miRs can be regulated and then targets downstream genes to re-sensitize cancer cells to the effects of DDP. For example, lidocaine alleviates DDP-resistance of MGC-803/DDP gastric cancer cells, inhibiting their migration through decreasing miR-10b expression (Zhang X. et al., 2020). Besides, the use nanoliposomes loaded with miR-1296 sensitizes breast cancer cells to DDP, by reducing CCND1, and thus, EMT process (Albakr et al., 2021). Finally, curcumin treatment is able to restrain the proliferation and facilitated apoptosis in HCT8/DDP cells, by promoting miR-497/Bcl-2 axis (Zheng ZH. et al., 2021). Consequently, it is just a matter of time until miR-based therapies be proved to restore the sensitivity of tumor cells to some anticancer drugs including DDP.

In this review, we have summarized some of our current understanding of microRNAs that affect DDP-resistance and some strategies that have been employed to sensitize cancer cells to DDP chemotherapy. These studies have improved our understanding of the involvement of miRs in drug resistance and provide a starting point for the development of ncRNA-based therapy to accelerate the resolution of DDP-resistance in many cancers, to improve the quality of life and prognosis of patients.
